# Interferon-α Inhibits NET Formation in Neutrophils Derived from Patients with Myeloproliferative Neoplasms in a Neutrophil Sub-Population-Specific Manner

**DOI:** 10.3390/ijms252413473

**Published:** 2024-12-16

**Authors:** Shirly Partouche, Idan Goldberg, Erez Halperin, Bahaa Atamna, Adi Shacham-Abulafia, Saar Shapira, Aladin Samara, Ayala Gover-Proaktor, Avi Leader, Galia Spectre, Pia Raanani, Galit Granot, Ofir Wolach

**Affiliations:** 1 Felsenstein Medical Research Center, Beilinson Hospital, Rabin Medical Center, Petah Tikva 4941492, Israel; 2 Institute of Hematology, Davidoff Cancer Center, Beilinson Hospital, Rabin Medical Center, Petah Tikva 4941492, Israel; idango@clalit.org.il (I.G.); piar@clalit.org.il (P.R.); 3 Faculty of Medical & Health Sciences, Tel Aviv University, Tel Aviv 6997801, Israel; 4 Department of Internal Medicine F—Recanati, Beilinson Hospital, Rabin Medical Center, Petah Tikva 4941492, Israel; 5 Hematology Service, Memorial Sloan Kettering Cancer Center, New York, NY 10065, USA; 6 Weill Cornell Medical College, New York, NY 10065, USA

**Keywords:** myeloproliferative neoplasia, neutrophil extracellular traps, high-density neutrophils, interferon-α

## Abstract

Neutrophils and neutrophil extracellular traps (NETs) contribute to thrombosis and hyperinflammation in myeloproliferative neoplasms (MPN). High-density neutrophils (HDNs) and low-density neutrophils (LDNs) have recently been characterized as distinct neutrophil sub-populations with distinct morphological and functional properties. We aim to study the kinetics of NET formation and inhibition with interferon-α (IFNα) in neutrophils derived from patients with MPN as compared to matched healthy controls. Ex vivo NET formation was assessed by neutrophil-elastase activity, neutrophil-associated nucleosomes, myeloperoxidase (MPO), and citrullinated histone H3 content. IFNα significantly inhibited NET formation in neutrophils derived from MPN patients. Neutrophil sub-population analysis demonstrated that HDNs drive the increase in NET formation as compared to LDNs in patients and in healthy controls and are effectively inhibited by IFNα, an effect that is lost in LDNs. In conclusion, we demonstrate that in MPN, HDNs drive excess NET formation and are more sensitive to IFNα inhibition. These observations uncover unique neutrophil sub-population biology and dynamics in MPN.

## 1. Introduction

Neutrophils are the most abundant myeloid cell type in circulation and are major effectors of the innate immune system [1]. In addition to their crucial role in fighting infections, neutrophils play a part in several pathological states via their contribution to inflammation, thrombosis, and tumorigenesis [2]. Recent evidence suggests that neutrophils are not a homogenous cell population in terms of morphology and function. In addition to a ‘normal’, abundant high-density neutrophil (HDN) sub-population, low-density neutrophils (LDNs) can be identified based on a characteristic density gradient centrifugation localization pattern [3,4]. LDNs were previously shown to exert pro-inflammatory properties and to promote cancer progression [5]. Expansion and activation of LDNs were observed in different pathologies including heart failure [6], autoimmune disorders [7,8,9], and cancer [5,10].

Myeloproliferative neoplasms (MPN) encompass a group of clonal stem-cell disorders characterized by increased blood counts, hyperinflammation, an arterial and venous thrombotic tendency, and varying risk for leukemic transformation [11]. Several studies highlight a pathogenic role for neutrophils in MPN [12]. An elevated neutrophil count and neutrophil-to-lymphocyte ratio were previously found to be associated with progression and thrombotic risk in these diseases [13,14,15]. Furthermore, neutrophils derived from patients with MPN and from MPN murine models were shown to possess an activated phenotype including an increase in neutrophil extracellular trap (NET) formation [16,17,18], a novel form of programmed cell death. In response to various infectious or metabolic stimuli, neutrophils can form NETs by expelling strands of decondensed DNA decorated with histones and different neutrophil granular proteins. These NET structures have the capability to ensnare and kill pathogens but are also associated with unwanted sequela such as thrombosis and autoimmunity [19,20]. Previous studies suggested that in MPN, NETs contribute to the thrombotic tendency, and inhibition of NET formation can abrogate the thrombotic risk [16]. The neutrophil sub-population-specific contribution to NET formation and to disease phenotype in MPN has not been previously addressed to the best of our knowledge.

Interferon-α (IFNα) is an effective cytoreductive treatment for patients with MPN and it was shown to reduce thrombotic complications in these disorders [21]. Furthermore, IFNα was suggested to reduce NET formation as assessed by plasma biomarkers of patients with MPN [22].

In the current study, we analyzed sub-population dynamics in neutrophils derived from patients with MPN. We focused on neutrophil sub-population-specific NET formation and response to IFNα therapy.

## 2. Results

### 2.1. Patient Characteristics

A total of 39 patients were enrolled in the study. The patients were divided into two distinct cohorts. The “General analysis” cohort included 23 patients, in whom we investigated the global effect of IFNα on NET formation in neutrophils derived from MPN patients compared to healthy controls. The “Density analysis” cohort consisted of 16 patients, where we categorized extracted neutrophils based on density (HDNs vs. LDNs) and examined how neutrophil density affected NET formation and response to IFNα exposure.

The clinical and laboratory characteristics of the patients are summarized in Table 1. Both cohorts were comparable in terms of baseline demographic and clinical characteristics. The median age was comparable between groups (51 [range: 42.5–60] years in the General analysis cohort and 53 [range: 41–71.2] years in the Density analysis cohort, *p* = 0.66). There were fewer males in the Density analysis cohort (*n* = 4, 25%) as compared to the General analysis cohort (*n* = 13, 56.5%. *p* = 0.051). The leading diagnosis for most patients in both groups was polycythemia vera (PV; 48% and 43.75% of patients, respectively) followed by essential thrombocytosis (ET; 34.8% and 25% of patients, respectively). *JAK2* V617F was the driving mutation in 18 patients (78.3%) in the General analysis cohort and in 14 patients (87.5%) in the Density analysis cohort.

### 2.2. IFNα Inhibits NET Formation in Neutrophils Derived from MPN Patients

**Figure 1:**
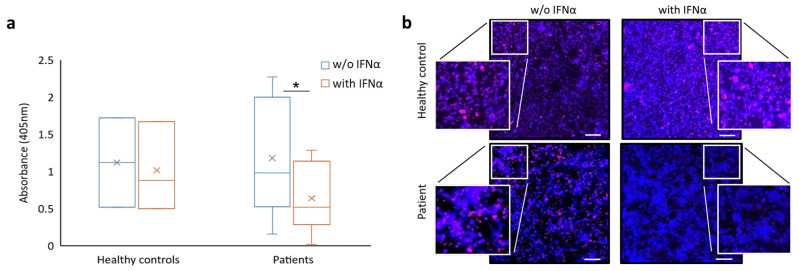
**IFNα decreases NET formation in neutrophils derived from patients with MPN.** (**a**) ELISA quantification of released nucleosomes by neutrophils from 10 MPN patients and from 3 healthy controls following activation with PMA with or w/o IFNα treatment. * *p* ≤ 0.03. (**b**) Confocal microscopy images of neutrophils from a representative PV patient and from a healthy control exposed to PMA with or w/o IFNα. Neutrophils were immunostained with anti-MPO (pink) and DAPI (blue). Magnification ×4. Scale bar = 100 μm.

Ex vivo NET formation was evaluated via neutrophil elastase activity in neutrophils stimulated with 100 nM phorbol 12-myristate 13-acetate (PMA) and treated or not with 5 ng/mL IFNα. Although basal NET generation was heterogeneous among patient samples, exposure to IFNα led to a marked reduction in neutrophil elastase activity in patient samples tested (*n* = 22). Average neutrophil elastase activity in neutrophils derived from patients was reduced from 22.32 ± 15.68 mU/mL to 18.56 ± 14.09 mU/mL after IFNα exposure (*p* ≤ 0.006). Interestingly, the reduction in neutrophil elastase activity was more prominent in PV patients compared to ET patients. Average neutrophil elastase activity in PV patient samples treated ex vivo with IFNα was 16.97 ± 12.24 mU/mL compared to 22.81 ± 15.03 mU/mL in the untreated samples (*p* ≤ 0.02; Table 2). In ET patients, the average initial elastase activity was 22.64 ± 17.45 mU/mL, and a more moderate decrease to 20.75 ± 16.95 mU/mL was noted following treatment with IFNα (not-significant, Table 2). In samples derived from ET patients, the reduction in NETosis markers was not influenced by the mutational status of *JAK2*. Conversely, neutrophil elastase activity in neutrophils derived from healthy controls (*n* = 10) was almost unaffected by IFNα treatment (Table 2). Consistent with the findings from the neutrophil elastase activity assay, the amount of extracellular DNA released by neutrophils (as assessed in the cell culture supernatants) decreased significantly in patients with MPN after these cells were exposed to IFNα (1.18 ± 0.74 vs. 0.63 ± 0.42, respectively; *p* ≤ 0.03; Figure 1a). Healthy controls did not exhibit such changes in extracellular DNA release (1.12 ± 0.6 vs. 1.01 ± 0.59, respectively; Figure 1a). Further supporting our results, immunofluorescent staining demonstrated a decrease in co-localization of DNA and MPO (a marker of NET formation) in neutrophil samples derived from MPN patients that were exposed ex vivo to IFNα (Figure 1b). Once again, healthy controls did not exhibit such changes in DNA and MPO co-localization (Figure 1b). The effect of IFNα on NET formation in neutrophils derived from patients with MPN was dose-dependent (Supplementary Materials Figure S1).

### 2.3. HDNs Are More Susceptible to NET Formation and Are Inhibited by IFNα

**Figure 2:**
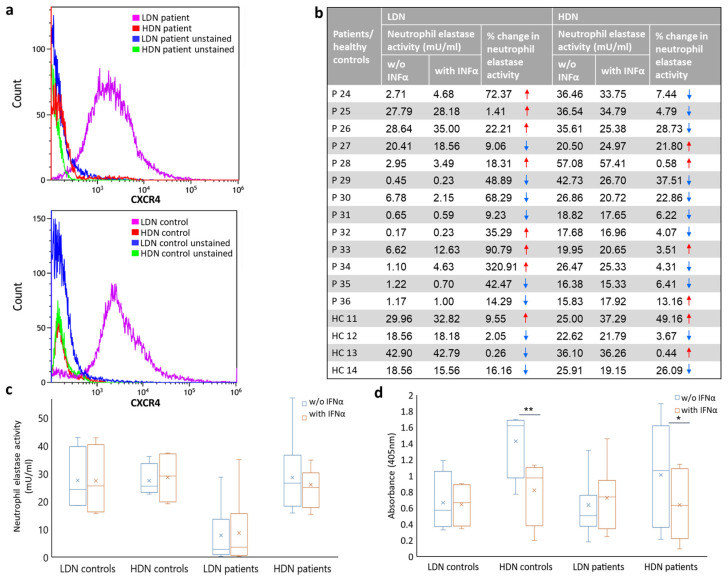
**HDNs are more susceptible to NET formation and are inhibited by IFNα.** (**a**) Expression of CXCR4, as measured by FACS analysis, in HDNs and LDNs from a representative PV patient (upper panel) and a healthy control (lower panel). (**b**) Change in neutrophil elastase activity in HDNs and LDNs derived from MPN patients (*n* = 13) and matched healthy controls (*n* = 4) following stimulation with PMA for 4 h with or w/o exposure to IFNα. Blue arrows designate a decrease and red arrows designate an increase in elastase activity following exposure to IFNα. (**c**) Neutrophil elastase activity in MPN neutrophil-sub-population (*n* = 13) compared with healthy controls neutrophil-sub-population quantified by ELISA. (**d**) ELISA quantification of released nucleosomes by HDNs and LDNs from MPN patients (*n* = 12) and from healthy controls (*n* = 4) with or w/o treatment of IFNα. * *p* ≤ 0.03, ** *p* ≤ 0.002.

We studied neutrophil sub-population dynamics of NET formation and the effect of IFNα exposure on these populations in neutrophils derived from MPN patients and controls. Initially, we isolated and characterized high- and low-density neutrophils from 13 MPN patients and from 4 age- and gender-matched healthy controls. Both LDNs and HDNs from patients and from healthy controls expressed high levels of CD11b and CD66b which are markers for an activated neutrophil state [23] (Supplementary Materials Figure S2). CXCR4, which was previously shown to be differentially expressed in LDNs as compared to HDNs [24], was also highly expressed in LDNs that we derived from both patients with MPN and controls as compared to HDNs (Figure 2a). In the patient samples, the percent of CXCR4-positive LDN and HDN cells was 90.5 ± 6.1 and 24.8 ± 1.7, respectively. In the control samples, the percent of CXCR4-positive LDN and HDN cells was 90.85 ± 0.75 and 42.85 ± 10.45, respectively. Next, we studied NET activation at baseline and following exposure to IFNα in LDNs and HDNs via measuring neutrophil elastase activity. At 4 h after stimulation with PMA (without IFNα exposure), HDNs from MPN patients showed higher baseline neutrophil elastase activity compared to LDNs from the same patient (28.53 ± 11.96 mU/mL and 7.74 ± 10.15 mU/mL, respectively; *p* ≤ 0.0003) (Figure 2b). Upon exposure to IFNα, a slight decrease in elastase activity was seen only in patient-derived HDNs (from 28.53 ± 11.96 mU/mL to 25.97 ± 10.78 mU/mL; Figure 2c). Healthy controls did not exhibit a decrease in elastase activity in LDNs or in HDNs (from 27.45 ± 10.03 mU/mL to 27.33 ± 11.08 mU/mL and from 27.40 ± 5.16 mU/mL to 28.62 ± 8.21 mU/mL, respectively; Figure 2c). The release of free DNA following PMA activation of neutrophils from 12 MPN patients and 4 healthy controls supported the observation of increased NET formation in HDNs compared to LDNs (1.01 ± 0.57 vs. 0.63 ± 0.41; *p* ≤ 0.05 in patients and 1.42 ± 0.38 vs. 0.65 ± 0.32 in healthy controls; *p* ≤ 0.01; Figure 2d). Incubation with IFNα resulted in a significant reduction in released DNA only in the HDN sub-population, which was reduced from 1.01 ± 0.57 to 0.63 ± 0.39 (*p* ≤ 0.03) in the patient samples and from 1.42 ± 0.38 to 0.81 ± 0.36 (*p* ≤ 0.002) in healthy controls (Figure 2d).

**Figure 3:**
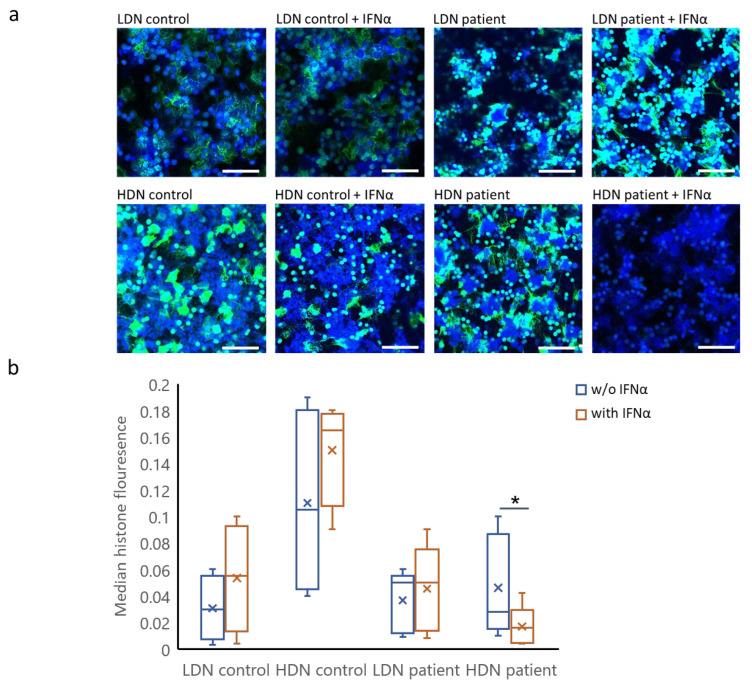
**IFNα reduces citrullinated histone H3 burden in HDNs derived from patients with MPN.** (**a**) Representative images of HDNs and LDNs derived from a patient with PV and a healthy control, exposed to PMA with and w/o IFNα. Neutrophils were immuno-stained with anti-histone H3 (citrulline R2 + R8 + R17, green) and with DAPI (blue). Magnification ×20. Scale bar = 100 μm. (**b**) Bar graphs showing the mean values of binary area fraction of citrullinated histone staining in HDNs and LDNs from patients and from healthy controls. The results are presented as the surface covered by citrullinated histone relative to the surface covered by cells (*n* =  6), * *p* ≤ 0.05.

To visualize and further confirm the differential effect of IFNα on NET formation in HDNs and LDNs, we assessed the burden of histone H3 citrullination, a hallmark of NET formation, by immunostaining neutrophils derived from patients and healthy controls. Confocal microscopy images of neutrophils stained for DNA (DAPI) and citrullinated histone H3 confirmed our observation that NET formation following activation with PMA is higher in HDNs (Figure 3a). Moreover, patient-derived HDNs treated with IFNα revealed a decrease in citrullinated histone H3 positivity, whereas LDNs did not (Figure 3a). Area fraction quantification of these results confirmed a significant reduction in citrullinated histone H3 staining in patient HDNs following exposure to IFNα (from 0.06 ± 0.03 to 0.01 ± 0.01; *p* ≤ 0.05; Figure 3b). No reduction in citrullinated histone H3 staining was observed in patient LDNs (from 0.04 ± 0.02 to 0.05 ± 0.02) or in healthy control HDNs (from 0.15 ± 0.03 to 0.11 ± 0.06) or LDNs (from 0.05 ± 0.03 to 0.03 ± 0.02; Figure 3b).

## 3. Discussion

Neutrophils are important effectors of disease phenotype in MPN and contribute to the hyperinflammation and thrombotic tendency that characterizes these diseases [25]. Neutrophils derived from patients with MPN were previously shown to be primed for NET formation [16,17]. In one analysis, MPO–DNA complex levels in the plasma of patients with MPN were increased as compared with controls, specifically in patients with a previous thrombotic event [18]. Neutrophils were also shown to be primed for NET formation in *JAK2*^V617F^ mutated murine models [16,26]. In these in vivo models, partial ligation of the inferior vena cava (IVC) resulted in higher rates of venous thrombosis in *JAK2*^V617F^ mice as compared to wild-type controls [26]. The mechanism by which NETs are increased in MPNs and contribute to the thrombotic risk are not completely understood. Moreover, this effect may be context dependent since not all studies were able to demonstrate these correlations [17,27]. Several observations suggest that neutrophil–platelet interactions [28,29,30], as well as cross-talk between neutrophils and the endothelial surface [31,32,33], play an important role in NET formation and thrombosis in these diseases.

Recent evidence suggests that neutrophils are highly plastic cells that display heterogeneous phenotypes with specific morphological and functional properties. Two distinct sub-populations have been identified: LDNs, which are thought to represent an immature, hyperactivated subtype of neutrophils, and HDNs [3,4,34]. Our analysis is the first, to our knowledge, to demonstrate the dynamics of NET production in neutrophil sub-populations in patients with MPN. We show that HDNs form significantly more NETs than LDNs in neutrophils derived from patients with MPN. This phenomenon does not seem to be merely a quantitative effect reflecting the higher abundance of HDNs in the blood of MPN patients since all assays used to assess NET formation were controlled for neutrophil counts. This finding is somewhat surprising given previous studies suggesting that LDNs are more prone to forming NETs in autoimmune and inflammatory conditions [6,35,36,37,38,39,40] and may reflect an ‘exhausted’ LDN phenotype in MPN. Several previous observations show that neutrophils derived from patients with MPN have an activated phenotype as assessed by immunophenotype and functional assays [16,33,41,42], although none have addressed a potential sub-population differential effect. In other malignant and inflammatory states, recent studies highlight the concept of differential, neutrophil sub-population-specific exhaustion of LDNs and increased NET formation in HDNs. Mauracher et al. studied neutrophil sub-population dynamics and NET formation in 20 patients with lung cancer as compared to controls. Patients with lung cancer had a higher proportion of activated LDNs compared to controls. However, only HDNs of patients with cancer had increased NET formation after ionomycin stimulation as compared to controls [43]. In a study of patients with antiphospholipid syndrome, LDNs from patients showed an exhausted phenotype while HDNs of patients were prone to activation and demonstrated higher NET production than HDNs from matched controls [44]. Cho et al. demonstrated similar results in neutrophils derived from patients with alcohol-associated hepatitis with functionally exhausted LDNs and activated HDNs prone for NET formation [45]. In our data set, we did not observe an over-activated phenotype of LDNs as assessed by the expression of the neutrophil activation markers CD11b and CD66b (Supplementary Materials Figure S2).

Interferons are cytokines with immunomodulatory properties and disease-modifying effects in MPN [21]. The development and clinical availability of the pegylated form (PEG) of IFNα established this therapeutic option as an effective and tolerable tool [46]. The exact mechanism of action by which IFNα reduces disease-related symptoms and thrombotic risk is still not fully understood. Massarenti et al. studied the effect of PEG-IFNα treatment on the serum levels of MPO-DNA complexes, a marker of NET formation, in patients with MPN enrolled on a clinical trial comparing PEG-IFNα therapy to hydroxyurea. PEG-IFNα-2a or PEG-IFNα-2b therapy were associated with reduced NET biomarker levels in 77% and 73% of patients, respectively, as compared to only 53% of patients treated with hydroxyurea [22]. Our analysis confirms the NET-inhibitory effect of IFNα and further demonstrates a neutrophil sub-population-specific effect, with inhibition of NET formation restricted to only the HDN population. The inhibitory effect of IFNα was more prominent in samples derived from patients with PV as compared to samples from patients with ET. The reason for this difference is unclear. This difference cannot be explained solely by the higher prevalence of *JAK2* mutations or by the higher allelic burden of *JAK2* mutations that is characteristic of patients with PV, as there was no significant association between the mutational status of *JAK2* and the reduction in NET formation markers following IFNα exposure in patients with ET. This observation is in line with previous observations that failed to demonstrate a significant correlation between *JAK2* mutational status and allelic burden and NET formation [16,17].

One potential limitation of this study is that the activation state of neutrophils, as well as NET formation, may be affected by several factors such as co-morbidities, medications, and neutrophil isolation procedures. To minimize this bias, we excluded patients that suffered from hyperinflammatory conditions that could affect NET formation and HDN/LDN dynamics (such as acute infection, active cancer, and autoimmune disease). Furthermore, all isolation procedures were centrally executed in a protocol-driven and similar manner, and three independent assays were employed to assess NET formation in the different samples. The gender imbalance in the Density analysis group (only 25% of patients in this group were male) may reflect the limited number of patients in this cohort and the PV diagnosis predominance. To minimize the potential effect of age and gender on NET formation, we used age- and gender-matched controls for each blood draw. The mechanism by which IFNα inhibits NET formation specifically in HDNs is unknown and should be the focus of future investigations.

In conclusion, we characterize neutrophil sub-populations in MPN. We show for the first time, to our knowledge, differential kinetics of NET formation and response to IFNα exposure in specific neutrophil sub-populations derived from MPN patients. We demonstrate that in MPN, HDNs drive excess NET formation and are more sensitive to IFNα inhibition. These observations uncover unique neutrophil sub-population biology and dynamics that can potentially be exploited for therapeutic purposes in the future.

## 4. Materials and Methods

### 4.1. Patients

Blood samples were obtained from adult patients with MPN and from age- and gender-matched healthy controls. All samples were obtained before any cytoreductive or JAK-inhibitor were started. Patients presenting with conditions known or suspected to alter NET formation were excluded from the study (including active or recent infection, active cancer, and autoimmune diseases). The study was approved by the local ethics committee, and all participants signed an informed consent prior to enrollment.

### 4.2. Neutrophil Isolation and Sub-Population Separation

Whole blood was collected into ethylenediaminetetraacetic acid (EDTA)-coated tubes. Neutrophils were isolated as previously described [47]. Briefly, 6 mL of whole blood was layered on top of 6 mL of histopaque (Biowest, Nuaillé, France) and centrifuged at 800× *g* for 20 min. The lower reddish phase containing granulocytes was transferred to a fresh tube, washed, resuspended in PBS, and layered on top of a homemade percoll gradient between 65–85% (Santa-Cruz, Dallas, TX, USA). The tubes were centrifuged at 800× *g* for 20 min. The top layer was removed, washed with PBS, and resuspended in 1 mL of NET assay buffer (Cayman Chemical, Ann Arbor, MI, USA). Neutrophil purity was ≥95%, as assessed by flow cytometry for all experiments. Neutrophil viability was ≥98%, as assessed by trypan blue staining.

Sub-populations of neutrophils were isolated using percoll gradient centrifugation, as previously described [48]. Briefly, 10 mL of whole blood was mixed with 10 mL of RPMI 1640 (Biowest) and then layered on top of 10 mL homemade percoll gradient between 62–75% (Santa-Cruz) and centrifuged at 200× *g* for 25 min and 400× *g* for 15 min. After centrifugation, layers containing peripheral blood mononuclear cells (PBMCs) and polymorphonuclear cells (PMNs) were isolated. Neutrophils within the PBMC layer were defined as LDNs. Neutrophils within the PMN layer were defined as HDNs. LDN and HDN layers were collected in separate tubes, washed once with RPMI 1640, and centrifuged at 300× *g* for 5 min, and the cells were resuspended in 1 mL of NET assay buffer (Cayman Chemical). Neutrophil viability was ≥98%, as assessed by trypan blue staining. The purity of the different sub-populations was evaluated by using antibodies directed against membrane markers, which are known to be differentially expressed in a specific sub-population. The purity of the HDN sub-population was assessed by staining with αCD11b and was found to be ≥99%. The purity of the LDN sub-population was assessed by staining with αCXCR4 and was found to be ≥92%.

### 4.3. Characterization of Neutrophil Surface Markers via Flow Cytometry

HDNs and LDNs (10^6^ cells/0.1 mL) from MPN patients and from healthy controls were incubated in PBS with antibodies of interest for 20 min at 4 °C in the dark. Antibodies used were mouse anti-human CD184 (CXCR4)-APC IgG2a antibody (BioLegend, San Diego, CA, USA, 5 µL/10^6^ cells), mouse anti-human CD11b-APC (SouthernBiotech, Birmingham, AL, USA, 5 µL/10^6^ cells), and mouse anti-human CD66b-FITC (SouthernBiotech, 5 µL/10^6^ cells). Cells were washed with PBS 3 times and analyzed using a flow cytometer (Gallios, Beckman Coulter, Indianapolis, IN, USA).

### 4.4. Short-Term Treatments and Activation of Neutrophils

Following isolation, neutrophils were seeded in 24-well plates at a concentration of 10^6^ cells/well. The cells were treated or not with 5 ng/mL recombinant human IFNα A (Alpha 2a) protein (PBL Assay Science, Piscataway, NJ, USA) [49], and NET formation was activated with 100 nM PMA (Cayman Chemical) for 4 h in RPMI 1640 medium containing 10% fetal bovine serum (FBS) at 37 °C in a humidified atmosphere with 5% CO_2_.

### 4.5. Cell Death Detection

A cell death ELISA kit (Roche, Rotkreuz, Switzerland) was used according to the manufacturer’s recommendations for quantification of released nucleosomes as surrogate markers of NETosis. In brief, after NETosis induction with PMA, 20 µL of supernatant per well was collected and transferred to an anti-histone-coated 96-well plate. Each well was then incubated with peroxidase-coupled anti-DNA antibody for 1.5 h at room temperature and then incubated with the ABTS peroxidase substrate solution on a shaker for ~20 min. Absorbance was measured at 405 nm using an ELISA plate reader (Epoch, BioTek, London, UK).

### 4.6. Neutrophil Elastase Activity

NET-bound neutrophil elastase was quantified using the Cayman NETosis assay kit (Cayman Chemical), according to the manufacturer’s instructions. Briefly, neutrophils were seeded in 24-well plates (10^6^ cells/well). Following stimulation with PMA to release NETs, unbound soluble neutrophil elastase was washed away. Then, S7 nuclease was added to each well and incubated for 45 min at 37 °C, to digest NET DNA. EDTA was then added to inactivate the nuclease, and the reactions were centrifuged for 5 min at 300× *g*. The supernatant containing neutrophil elastase was transferred to new wells and elastase substrate was added and incubated for 1 h at 37 °C. Absorbance was then measured at 405 nm using an ELISA plate reader (Epoch, BioTek).

### 4.7. Immunostaining, Confocal Microscopy and NET Quantification

Neutrophils were plated on glass coverslips and placed in 24-well plates at 37 °C in 5% CO_2_. Following NETosis stimulation, the cells were washed with PBS and then fixed with 4% paraformaldehyde for 60 min at room temperature. The fixed neutrophils were permeabilized with 0.2% Triton X-100 and 0.1% sodium citrate for 10 min at 4 °C. The supernatant was discarded, slide chambers were washed with PBS, and cells were blocked with 1% bovine serum albumin at 37 °C for 30 min before staining with rabbit polyclonal anti-histone H3 (citrulline R2 + R8 + R17, Abcam, Trumpington, Cambridge, UK) or mouse anti-human MPO (Abcam) at 4 °C overnight. For secondary antibody staining, goat anti-rabbit IgG (H + L) Cross-Adsorbed Ready Probes^TM^ secondary antibody, Alexa Fluor™ 488 (ThermoFisher, Waltham, MA, USA) and goat anti-mouse IgG secondary antibody, DyLight™ 680 (ThermoFisher) were used. DNA was counterstained with 4′,6-diamino-2-phenylindole, dihydrochloride (DAPI) (Milteny Biotec, San Jose, CA, USA). Coverslips were washed 3 times with PBS and mounted on slides with ProLong^TM^ gold antifade reagent. Images were captured and analyzed by confocal microscopy (Nikon AXR Confocal Microscope System, Nikon, Tokyo, Japan). To quantify NETs area, the number of green pixels (i.e., surface covered by histones colored by Alexa Fluor™ 488 green) and blue pixels (surface covered by neutrophils, colored by DAPI) were measured using NIS Elements C software Version 5.3 (Nikon) with a threshold to exclude background fluorescence. Percentage of histone staining was determined from five nonoverlapping fields per well for every sample. The results are presented as the binary fraction area, which represents the surface covered by histone staining relative to the surface covered by cells.

### 4.8. Statistical Analysis

Patient characteristics were summarized by standard descriptive statistics. Continuous variables were described by median values and corresponding interquartile range (IQR). Wilcoxon−Mann−Whitney-U-test was used to compare differences between independent groups. A two-sided *p* ≤ 0.05 was defined as the threshold for statistical significance. Statistical analysis was performed with the commercially available package SAS, version 9.4 (SAS Institute, Cary, NC, USA). When distribution of data is shown in box plots, the boxes indicate medians, as well as 25% and 75% quartiles; whiskers represent the expected variation of the data. The whiskers extend 1.5 times the IQR from the top and bottom of the box.

## Figures and Tables

**Table 1:** Baseline characteristics of the study patients.

Parameter *	General Analysis ^#^ *n* = 23	Density Analysis ^†^ *n* = 16	*p*-Value
**Age (years)**	51 (42.5–60)	53 (41–71.25)	0.66
**Male (n, (%))**	13 (56.5)	4 (25)	0.051
**Type of MPN**			0.57
**PV (n, (%))**	11 (47.8)	7 (43.75)	
**ET (n, (%))**	8 (34.8)	4 (25.0)	
**Unclassifiable (n, (%))**	4 (17.4)	5 (31.25)	
**Mutational status**			0.58
***JAK2*** **V617F (n, (%))**	18 (78.0)	14 (87.5)	
***JAK2*** **exon 12 (n, (%))**	1 (4.3)	1 (6.25)	
**CALR (n, (%))**	4 (17.4)	1 (6.2)	
**MPL (n, (%))**	0 (0)	0 (0)	
**Vasomotor symptoms (n, (%))**	5 (21.7)	6 (37.0)	0.28
**Enlarged spleen (n, (%))**	8 (34.8)	6 (37.5)	0.86
**Thrombotic/hemorrhagic complications**			
**Low-risk disease (n, (%))** **^‡^**	18 (78.3)	11 (68.75)	0.9
**ATE (n, (%))**	0	0	
**VTE (n, (%))**	0	0	
**Bleeding events (n, (%))**	1 (4.3)	0	0.398
**Aspirin treatment (n, (%))**	17 (73.9)	14 (87.5)	0.3
**Anticoagulation treatment (n, (%))**	0 (0)	1 (6.25)	0.22
**Hemoglobin (g/dL)**	14.4 (13.2–15.05)	13.7 (13.17–15.72)	0.92
WBC (* 10^9^/L)	7.32 (6.59–9.18)	8.8 (7.5–11.5)	0.09
Platelet count (* 10^9^/L)	570 (486–710)	426 (383–510)	0.11

* Values are in median (IQR). ^#^ In the “General analysis” cohort, we investigated the effect of IFNα on NETosis in neutrophils derived from MPN patients compared to healthy controls. ^†^ In the “Density analysis” cohort, we categorized extracted neutrophils based on density (HDNs vs. LDNs) and examined how neutrophil density affected NETosis in response to IFNα exposure. ^‡^ The definition of low-risk PV patients is based on the European Leukemia Net 2018 guidelines. The definition of low-risk ET patients is based on the revised IPSET criteria. In this table, the term ‘low-risk’ refers to both patients with low-risk disease and very-low-risk disease according to the revised IPSET criteria. We implemented the same definition for patients with unclassifiable MPN disease. ATE = arterial thromboembolism; CALR = calreticulin; ET = essential thrombocytosis; *JAK2* = Janus kinase 2; MPL = myeloproliferative leukemia virus oncogene; MPN = myeloproliferative neoplasm; PV = polycythemia vera; VTE = venous thromboembolism; WBC = white blood cells.

**Table 2:** Neutrophil elastase activity in neutrophils from MPN patients and healthy controls *.

Patients/ Healthy Controls	Neutrophil Elastase Activity w/o IFNα (mU/mL)	Neutrophil Elastase Activity with IFNα (mU/mL)	% Change in Neutrophil Elastase Activity	Type of MPN
P 01	34.37	10.20	70.32 ↓	PV
P 02	19.58	19.65	0.36 ↑	NOS
P 03	2.04	1.97	3.43 ↓	PV
P 04	3.09	6	94.17 ↑	ET
P 05	8.26	5.44	34.14 ↓	PV
P 06	11.96	11.4	4.68 ↓	PV
P 07	4.26	2.45	42.49 ↓	PV
P 08	8.71	7.29	16.30 ↓	NOS
P 09	3.59	2.61	27.30 ↓	ET
P 10	5.57	5.47	1.80 ↓	ET
P 11	3.85	2.59	32.73 ↓	PV
P 12	35.85	30.76	14.20 ↓	PV
P 13	35.21	31.68	10.03 ↓	PV
P 14	54.09	51.91	4.03 ↓	ET
P 15	18.35	8.57	53.30 ↓	ET
P 16	44.90	29.82	33.59 ↓	PV
P 17	23.14	21.76	5.96 ↓	ET
P 18	30.73	28.63	6.83 ↓	NOS
P 19	36.97	35.27	4.60 ↓	ET
P 20	36.33	34.43	5.23 ↓	ET
P 21	35.06	31.44	10.33 ↓	PV
P 22	35.03	28.95	17.36 ↓	PV
HC 01	2.59	2.57	0.77 ↓	
HC 02	4.07	4.04	0.74 ↓	
HC 03	4.80	4.27	11.04 ↓	
HC 04	39.47	40.30	2.10 ↑	
HC 05	21.65	21.73	0.37 ↑	
HC 06	22.35	22.10	1.12 ↓	
HC 07	36.60	37.13	1.45 ↑	
HC 08	31.20	32.53	4.26 ↑	
HC 09	34.13	34.43	0.88 ↑	
HC 10	37.00	37.97	2.62 ↑	

* Neutrophil elastase activity in neutrophils derived from 22 patients (P) with MPN and 10 healthy controls (HC) exposed to PMA and treated or not with IFNα. **↓** denote a decrease and **↑** denote an increase in elastase activity following exposure to IFNα.

## Data Availability

All data generated and analyzed during this study are available from the corresponding author upon reasonable request.

## Supplementary Materials

The following supporting information can be downloaded at: https://www.mdpi.com/article/10.3390/ijms252413473/s1.
