# Supplementary material for: Interferon-α Inhibits NET Formation in Neutrophils Derived from Patients with Myeloproliferative Neoplasms in a Neutrophil Sub-Population-Specific Manner
Source: Int J Mol Sci. 2024 Dec 16;25(24):13473. doi: 10.3390/ijms252413473 (PMC11677445; doi:10.3390/ijms252413473)
Supplement: Supplementary file 1 [file ijms-25-13473-s001.zip › ijms-3327343-supplementary.pdf]

International Journal of
Molecular Sciences

Article

Interferon-α Inhibits NET formation in Neutrophils Derived from Patients with Myeloproliferative Neoplasms in a Neutrophil Sub-population-Specific Manner

Partouche, Goldberg, et al.

Supplementary Materials

Figure S1: INFα decreases NET formation in neutrophils derived from patients with MPN in a dose dependent manner.

Figure S2: CD11b and CD66b expression on neutrophils.

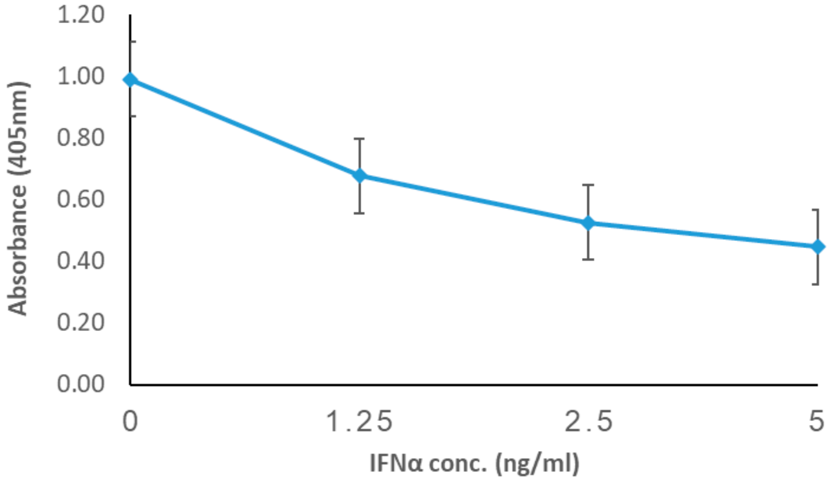

Figure S1. INFα decreases NET formation in neutrophils derived from patients with MPN in a dose dependent manner. ELISA quantification of released nucleosomes by neutrophils from MPN patients (n=2) following treatment with INFα at different concentrations.

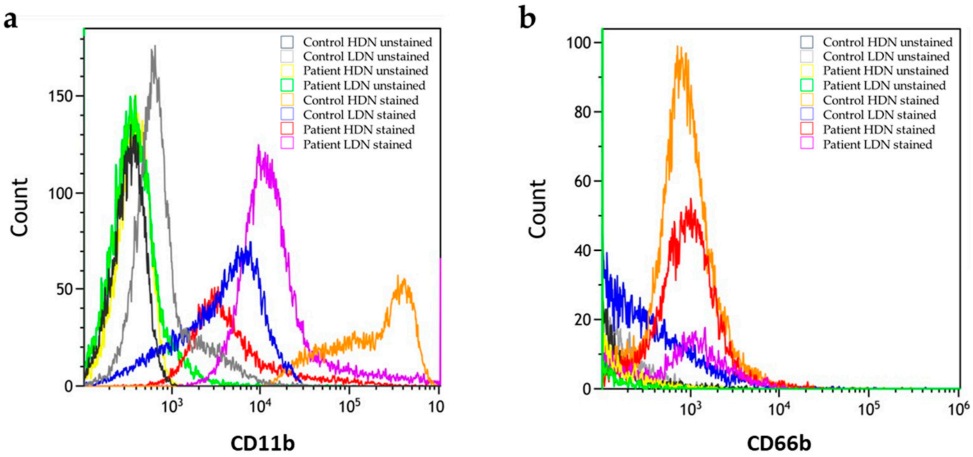

Figure S2. **CD11b and CD66b expression on patient neutrophils.** Representative histograms depicting the expression of CD11b and CD66b in HDNs and LDNs from a representative PV patient and a healthy control, as analyzed by flow cytometry. Black, grey, yellow and green lines represent unstained samples; orange and red lines represent control and patient HDN samples, respectively; and blue and purple lines represent control and patient LDN samples, respectively. Representative data out of three independent experiments.
